# Matrix Metalloproteinase 10 Contributes to Choroidal Neovascularisation

**DOI:** 10.3390/biomedicines10071557

**Published:** 2022-06-30

**Authors:** Jorge González-Zamora, María Hernandez, Sergio Recalde, Jaione Bezunartea, Ana Montoliu, Valentina Bilbao-Malavé, Josune Orbe, José A. Rodríguez, Sara Llorente-González, Patricia Fernández-Robredo, Alfredo García-Layana

**Affiliations:** 1 Retinal Pathologies and New Therapies Group, Experimental Ophthalmology Laboratory, Department of Ophthalmology, Clinica Universidad de Navarra, 31008 Pamplona, Spain; jgzamora@unav.es (J.G.-Z.); srecalde@unav.es (S.R.); jbezunartea@unav.es (J.B.); amontoliu@unav.es (A.M.); vbilbao@unav.es (V.B.-M.); sllorente@unav.es (S.L.-G.); aglayana@unav.es (A.G.-L.); 2 Navarra Institute for Health Research, IdiSNA, 31008 Pamplona, Spain; josuneor@unav.es (J.O.); josean@unav.es (J.A.R.); 3 Laboratory of Atherothrombosis, Program of Cardiovascular Diseases, CIMA-Universidad de Navarra, CIBERCV, 31008 Pamplona, Spain

**Keywords:** matrix metalloproteinase, age-related macular degeneration, oxidative stress, choroidal neovascularisation, angiogenesis

## Abstract

Age-related macular degeneration (AMD) is currently the main cause of severe visual loss among older adults in developed countries. The pathophysiology has not been clarified, but oxidative stress is believed to play a major role. Matrix metalloproteinases (MMP) may play a prominent role in several steps of the pathophysiology of AMD, especially in its neovascular form; therefore, there is of great interest in understanding their role in choroidal neovascularisation. This study aimed to elucidate the role of MMP10 in the development of choroidal neovascularisation (CNV). We have demonstrated that MMP10 was expressed by retinal pigment epithelium cells and endothelial cells of the neovascular membrane, in cell culture, mouse and human retina. MMP10 expression and activity increased under oxidative stress conditions in ARPE-19 cells. MMP10^-/-^ mice developed smaller laser-induced areas of CNV. Furthermore, to exclude a systemic MMP10 imbalance in these patients, plasma MMP10 concentrations were assessed in an age- and sex-matched sample of 52 control patients and 52 patients with neovascular AMD and no significant differences were found between the groups, demonstrating that MMP10 induction is a local phenomenon. Our findings suggest that MMP10 participates in the development of choroidal neovascularisation and promotes MMP10 as a possible new therapeutic target.

## 1. Introduction

Prolonged life expectancy leads to the increasing prevalence of age-related macular degeneration (AMD), which is one of the leading causes of severe vision loss in the elderly in developed countries [1]. Approximately 30–50 million people worldwide are affected by AMD [2]. In addition to blindness, AMD-related deterioration in visual function affects patients’ quality of life and can lead to loss of independence, social isolation, and absenteeism.

AMD is currently classified into the following stages: early stage, characterized by drusen and pigmentary changes; intermediate stage, characterized by the presence of large drusen, retinal pigment epithelium (RPE) cells abnormalities, or both; and late stage, divided into two subtypes—geographic atrophy (GA), also known as the dry form, and choroidal neovascularisation (CNV), also known as the wet form [3]. The two late stages are morphologically very distinct, although the early stages are indistinguishable. It is unclear why some AMD patients who progress to the late stage of the disease develop CNV, while others develop GA.

With age, the extracellular matrix (ECM) undergoes significant changes, affecting its function, leading to the accumulation of waste material [4]. Drusen formation, a hallmark of AMD, is thought to be due to RPE cell dysfunction and dysregulated ECM remodelling [4]. The structural and functional properties of the ECM are maintained through closely regulated processes of synthesis and degradation. Activated matrix metalloproteinases (MMPs) can digest all components of the ECM, such as elastin; gelatin; and collagen I, IV, and V, and orchestrate continuous remodelling of the membrane [5].

Oxidative stress is one of the main causes leading to ageing and consequently it has been found to play a central role in many neurodegenerative diseases, including AMD [6] and a major factor promoting MMP expression and activity is oxidative stress [7].

In humans, the MMP family is composed by 23 proteins, among which at least 14 are expressed in the human vasculature [8]. Since MMPs can interact with a variety of substrates, such as cytokines, cell surface molecules, or non-ECM molecules [9], they are involved in various processes, including proteolysis, cell adhesion, angiogenesis, wound healing, inflammation, cell proliferation, and development processes [10].

MMP2 and MMP9 are the most studied MMPs in relation to ocular neovascular pathology [11]. The role of other MMPs is unknown, although many of them have been linked to neovascularisation processes in several organs [12]. MMP10 has been associated with inflammatory pathology, wound healing, and vascular remodelling [13,14,15,16,17]. In ocular vascular pathology, elevated vitreous concentrations of MMP10 have been described in patients with venous occlusions with ischaemic manifestations [18], and the plasma levels correlate with vascular complications in patients with diabetic retinopathy [16,19].

In this study, we explored the possible role of MMP10 in the development of CNV at the cellular, animal, and human levels. To achieve these objectives, we characterised MMP10 expression in the RPE under oxidative stress in mouse and human retina. We further analysed the role of MMP10 in the development of CNV in an MMP10-deficient mouse model of laser-induced CNV. Finally, we examined the plasma levels of MMP10 in patients with wet AMD.

## 2. Materials and Methods

### 2.1. Cell Culture

#### 2.1.1. ARPE-19 Cell Culture

ARPE-19 were obtained from American Type Culture Collection (ATCC) (CRL-2302, Manassas, VA, USA), (p12, p14 and p16) were grown to confluence in a 37 °C incubator with humidified of, 5% CO_2_ in air in Dulbecco’s modified Eagle’s medium (DMEM; D6429, Sigma-Aldrich, St. Louis, MO, USA) containing 10% FBS; 10270106 Gibco ThermoFisher, Paisley, UK), 1% fungizone (Gibco, Carlsbad, CA, USA), and penicillin–streptomycin (Gibco, Carlsbad, CA, USA). Three times per week the culture medium was replaced. Once they reached confluence, the medium was changed to 1% fetal bovine serum (FBS) and replaced with the same frequency up to 2.5 months.

#### 2.1.2. Primary Human RPE Cell Culture

Three human donor eyes were used to isolate human RPE (hRPE) cells. Donations were acquired through the Department of Pathology, Anatomy and Physiology of the School of Medicine, University of Navarra. All procedures have been performed in accordance with the principles stated in the Declaration of Helsinki and local ethical committee.

For microbial decontamination eyes were immersed in povidone-iodine (10%) for two minutes. Then, they were cut below the ora serrata, and the posterior eyecup was separated. The neural retina was replaced from the posterior mice eyecup and the RPE/choroid tissue was maintained for 3 days in complete DMEM:F-12 medium (ATCC, Manassas, VA, USA). Then, RPE cells were softly dislodged by brushing with a fire-polished glass spatula. The dislodged RPE cells were centrifuged at 200 *g* for 10 min and the pellet was suspended in DMEM:F-12 (ATCC, Manassas, VA, USA) medium supplemented with 10% FBS and 1% penicillin/streptomycin/amphotericin B (Lonza; complete medium, Basel, Switzerland), plated into 24-well tissue culture plates and grown to confluence in a standard incubator at 37 °C under humidified 5% CO_2_ conditions. After proliferation, the hRPE cells were trypsinized and cultured in the same conditions and the culture medium changed twice a week. Passages 1 to 3 were used in the study.

#### 2.1.3. Induced Oxidative Stress Conditions

ARPE-19 cells were plated in 24-well plates (Corning Life Science, Tewksbury, MA, USA) and cultured for 2.5 months as previously explained. Then, cells were subjected to H_2_O_2_ (800 µM, Panreac, Barcelona, Spain) for 6, 24, 30 and 48 h. Supernatants and lysates were obtained at each timepoint. MMP10 concentration was analyzed by western blot (WB).

The effect of H_2_O_2_ on MMP10 expression was evaluated by immunofluorescence in ARPE-19 monolayers cultured on transwell inserts and coverslips (three passages were used, p12, 14 and 16). To confirm the phenotype zonula occludens-1 (ZO-1) and cytokeratin 18 (CK18) immunofluorescence was performed. One-hundred thousand ARPE-19 cells per well were seeded on laminin-coated polycarbonate membrane in Transwell cell culture inserts (Corning Life Science, Tewksbury, MA, USA). The cells were seeded at a density of 50 × 10^3^/coverslip. ARPE-19 cells were grown in 1% FBS-DMEM for 2.5 months. Cells were fixed in cold methanol overnight at −20 °C and washed with 1% phosphate buffer saline (PBS) and then incubated with blocking buffer containing 1% bovine serum albumin (BSA), 0.5% Triton X-100, 0.2% sodium azide, and 1% FBS for 1 h at 4 °C. Cells were incubated with the following antibodies: ZO-1 Alexa Fluor 594 mouse monoclonal antibody (1:100, 339194, Invitrogen-Life Technologies, Gaithersburg, MD, USA), CK18 mouse monoclonal antibody (1:250, M7010, DAKO, Glostrup, Denmark) and rabbit polyclonal antibody to MMP10 (1:250, OAAI00209, Aviva, San Diego, USA), diluted in blocking buffer at 4 °C for 24 h. The cells were washed three times each for 10 min and then incubated with the secondary fluorescent antibodies goat anti-mouse 488 (1:250, A11029, Life technologies, Gaithersburg, MD, USA) and donkey anti-rabbit 594 (1:250, R37119, Invitrogen, Carlsbad, CA, USA) diluted in blocking buffer during 1 h in the dark. Nuclei were stained with 4′,6-diamidino-2-phenylindole (DAPI; Sigma-Aldrich, St. Louis, MO, USA). Images were obtained with a laser scanning confocal imaging system (LSM800, Zeiss, Oberkochen, Germany).

#### 2.1.4. Western Blotting for MMP10

MMP10 protein was quantified from ARPE-19 and hRPE lysates and cell culture supernatants. Equal amounts of RPE homogenate (3.5 µg) were mixed with Laemmli buffer (4× NuPage, Invitrogene, Carlsbad, CA, USA) and boiled for 5 min. Samples were separated in 12% sodium dodecyl sulfate polyacrylamide gel electrophoresis (SDS-PAGE) gels and transferred to nitrocellulose membranes (GE Healthcare, Fairfield, CT, USA). Then, samples were blocked with 5% skimmed milk (*w*/*v*), 0.1% Tween-20 (*w*/*v*) in Tris buffer saline (TBS) (1 h, RT) and membranes were exposed to anti-MMP10 Rabbit Polyclonal Antibody (1:1000, TA321484, OriGene, Maryland, USA) overnight at 4 °C. Membranes were then incubated with a horseradish peroxidase conjugated goat anti-rabbit antibody (7074S; 1:5000, Cell Signaling Technology Inc., Danvers; MA, USA). Chemoluminescent signals were detected with an enhanced chemoluminescence (ECL) kit (ECL-Advance™ Western Blotting Detection Kit, GE Healthcare, Fairfield, CT, USA) and signals were measured using the ImageQuant 400 software (GE Healthcare, Fairfield, CT, USA). The relative intensities of immunoreactive bands were quantified with ImageQuant TL software (GE Healthcare, Fairfield, CT, USA).

#### 2.1.5. RQ-PCR of MMP10 mRNA

Relative quantification analysis was conducted to confirm the expression of MMP10 (Hs00233987_m1) using Taqman expression assays. The expression of this gene was determined in ARPE-19 cells subjected to H_2_O_2_ (800 µM, Panreac, Barcelona, Spain; for 2 h, 6, 8 and 24 h) and compared to controls (1% FBS). ABI PRISM™ 6100 Nucleic Acid PrepStation (Life Technologies, Carlsbad, CA, USA) were used to obtain mRNA from ARPE-19. Using the qScript cDNA Supermix Kit (Quanta Biosciences, Gaithersburg, MD, USA), 500 ng of each mRNA was reverse transcribed using a 2720 Thermal Cycler (Life Technologies, Carlsbad, CA, USA). The 7300 Real-Time PCR System (Life Technologies, Carlsbad, CA, USA) was used for amplification, and two housekeeping genes (glyceraldehyde 3-phosphate dehydrogenase and ß-actin) were used as internal controls.

### 2.2. Laser-Induced Choroidal Neovascularisation Model in Mice

All experiments were performed in accordance with the European Community guidelines for ethical animal care and use of laboratory animals (Directive 2010/63/UE) and were approved by the University of Navarra Animal Research Review Committee (166-12).

Thirteen WT males (12–16 weeks-old, C57Bl6/J, Charles River, Wilmington, MA, USA) and eight age-matched MMP10-deficient mice were provided by Dr. Josune Orbe (CIMA-Universidad de Navarra, Pamplona, Spain) (MMP10^-/-^, C57BL/6 background were housed in standard cages with 12-h light/dark cycle and food and water provided ad libitum.

Briefly, mice were anesthetized with xylazine (10 mg/kg; Xilagesic 2%; Calier Laboratories, Barcelona, Spain) and ketamine (75 mg/kg; Imalgene 1000; Merial Laboratories, Barcelona, Spain). Pupils were dilated with tropicamide (3 mg/mL; Alcon Cusí) and phenylephrine (7.8 mg/mL; Alcon Cusí, Barcelona, Spain) eye drops. CNV lesions were performed by using a 532-nm laser (Micron IV, Phoenix Research Laboratories). Three to four laser photocoagulation spots (250 mW intensity, 0.05 s and 50 µm spot size) were created concentrically around the optic nerve. Development of a bubble confirmed the traumatic rupture of Bruch’s membrane (BM). Laser spots with vitreous hemorrhage or lacking bubble at the time of laser application were excluded from analysis, which occurred in <10% of each group.

### 2.3. Neovascularisation Staining and MMP10 Immunofluorescence

After euthanasia, mice eyes were enucleated and fixed for 1 h in 4% paraformaldehyde diluted in PBS at 4 °C. RPE-choroid-sclera complex was microsurgically isolated, flat mounted and immersed with blocking buffer containing PBS, 3% Triton X-100, 0.5% Tween 20, 2% sodium azide, and 1% FBS for 1 h at 4 °C. Then, they were incubated with biotinylated isolectin (1:240, B-1205; Vector Labs, Burlingame, CA, USA) or rat anti-CD31 (1:50; DIA-310, Dianova, Hamburg, Germany), and rabbit polyclonal antibody to MMP10 (1:250, OAAI00209, Aviva, San Diego, USA) overnight at RT for 24 h, and the flatmounts were washed in PBS. Samples were incubated with donkey anti-rat 594 (1:250; S32356; Life Technologies, Carlsbad, CA, USA) and donkey anti-rabbit 647 (1:250, A31573, Invitrogen, Massachusetts, USA) and streptavidin Alexa Fluor 488 (1:250; S32354; Thermo ThermoFisher, Paisley, UK). The CNV lesions were captured under fluorescence and confocal microscopes (Axio Imager M1 and LSM800; Zeiss, Oberkochen, Germany), and the areas were measured by Fiji ImageJ software by two independent investigators.

### 2.4. Human Retina Immunofluorescence

#### 2.4.1. Human Tissue Processing for MMP10 Immunofluorescence

Human donor eyes (*n* = 3) were used in this study for retinal flat mount immunofluorescence. The retinas showed no apparent macroscopic changes such as disciform scars, hemorrhages, or pigmentary changes. The eyes were fixed in 4% paraformaldehyde diluted in phosphate buffer (PB) for 3 h and 2% paraformaldehyde for 6 days at 4 °C. Then, the eye was cryopreserved in 15% sucrose 24 h and 30% sucrose until use. RPE and retinas were removed from optic cup, washed in phosphate buffer saline (PBS) and flatmounted.

#### 2.4.2. Immunofluorescence in Human Flatmounted Eyes

Retinal and RPE flatmounts were incubated in blocking buffer mentioned above for 5 h at 4 °C. Then, they were subjected to rabbit polyclonal antibody to MMP10 and biotinylated isolectin (references above) for 3 days at RT. Flatmounts were washed in PBS and incubated in Alexa Fluor goat anti-rabbit 594 for 3 h, streptavidin Alexa Fluor 488 (1:250; S32354; Thermo ThermoFisher, Paisley, UK) and Hoescht (H1399, ThermoFisher, Waltham, MA, USA) and 4′,6-diamidino-2-phenylindole (DAPI) (Sigma-Aldrich, St. Louis, MO, USA). Flatmounts were mounted with PBS-glycerol (1:1) and visualized under a confocal microscope (LSM800, Zeiss, Oberkochen, Germany).

### 2.5. WetAMD Patients

#### 2.5.1. Patient Sample

Our study included 52 patients with wet AMD (AREDS category 4) and age- and sex-matched 52 control participants (AREDS category 1) from 2 tertiary referral hospitals: Clínica Universidad de Navarra and Hospital Universitario de Navarra. All participants were of Caucasian origin and gave written informed consent. All procedures were performed in accordance with the ethical standards of the Institutional Ethics Review Board of the Clínica Universidad de Navarra (Protocol: 2016.092mod1) and with the 1964 Helsinki Declaration and its later amendments, or comparable ethical standards. Inclusion criteria for patients with wet AMD included the following: diagnosis of AMD with active subfoveal or juxtafoveolar CNV confirmed by fluorescein angiography (FA) and/or optical coherence tomography (OCT) (AREDS category 4). For control participants, inclusion criteria were the following: absence of drusen or no more than 5 small drusen (≤63 μm), absence of retinal pigment abnormalities in the macular area, and absence of chorioretinal macular atrophy or any other form of CNV (AREDS category 1). Exclusion criteria for this study (for both patients with wet AMD and control participants) included: age younger than 55 years, the presence of other CNV-related retinal diseases (i.e., angioid streaks, nevus in the macular area, toxoplasmosis scars, photocoagulation scars in the posterior pole, or central serous chororetinopathy), history of retinal surgery, retinal disease in the studied eye (i.e., diabetic retinopathy or hereditary retinal dystrophies), and more than 6 diopters of myopia. All cases underwent detailed ophthalmologic examination, including visual acuity assessment, dilated slit-lamp biomicroscopy, automatic objective refraction, color fundus photography, FA, and/or OCT. Controls underwent visual acuity assessment, mydriatic fundus examination, and measurement of refractive error and axial length.

#### 2.5.2. Plasma Analysis

For plasma collection, 6 mL venous blood samples were obtained from peripheral blood in Ethylenediaminetetraacetic acid (EDTA) containing tubes and centrifuged at 1000 g for 15 min at room temperature (RT). The plasma was then transferred to another tube within the first hour of extraction and the samples were frozen at −80 °C until used.

#### 2.5.3. MMP10 Measurements by ELISA

Levels of MMP10 in plasma samples were assessed in duplicate by enzyme-linked immunosorbent assay (ELISA) according to the manufacturer’s instructions (DM1000, R&D Systems, Abingdon, UK; dilution 1:10). The detection limit for MMP10 was 78.1 pg/mL. Inter- and intra-coefficients of variation were <8%.

### 2.6. Statistical Analysis

The Kolmogorov–Smirnov test was used to evaluate the normality of the distributions. Quantitative variables following a normal distribution were compared with the independent Student’s *t*-test. For non-normally distributed variables, the Mann-Whitney U test were used. For correlations between variables, Pearson’s correlation coefficient was used for parametric distributions and Spearman’s correlation coefficient for non-parametric ones. For all tests, *p* values < 0.05 were considered statistically significant.

Data are presented as mean ± SEM. Statistical analysis was performed using GraphPad Prism 8.0 (GraphPad Software, San Diego, CA, USA) software.

## 3. Results

### 3.1. MMP10 Expression Pattern and Oxidative Stress Effect on Transcription, Synthesis and Activation of MMP10

#### 3.1.1. MMP10 Is Expressed in ARPE-19 and hRPE Cells

**Figure 1:**
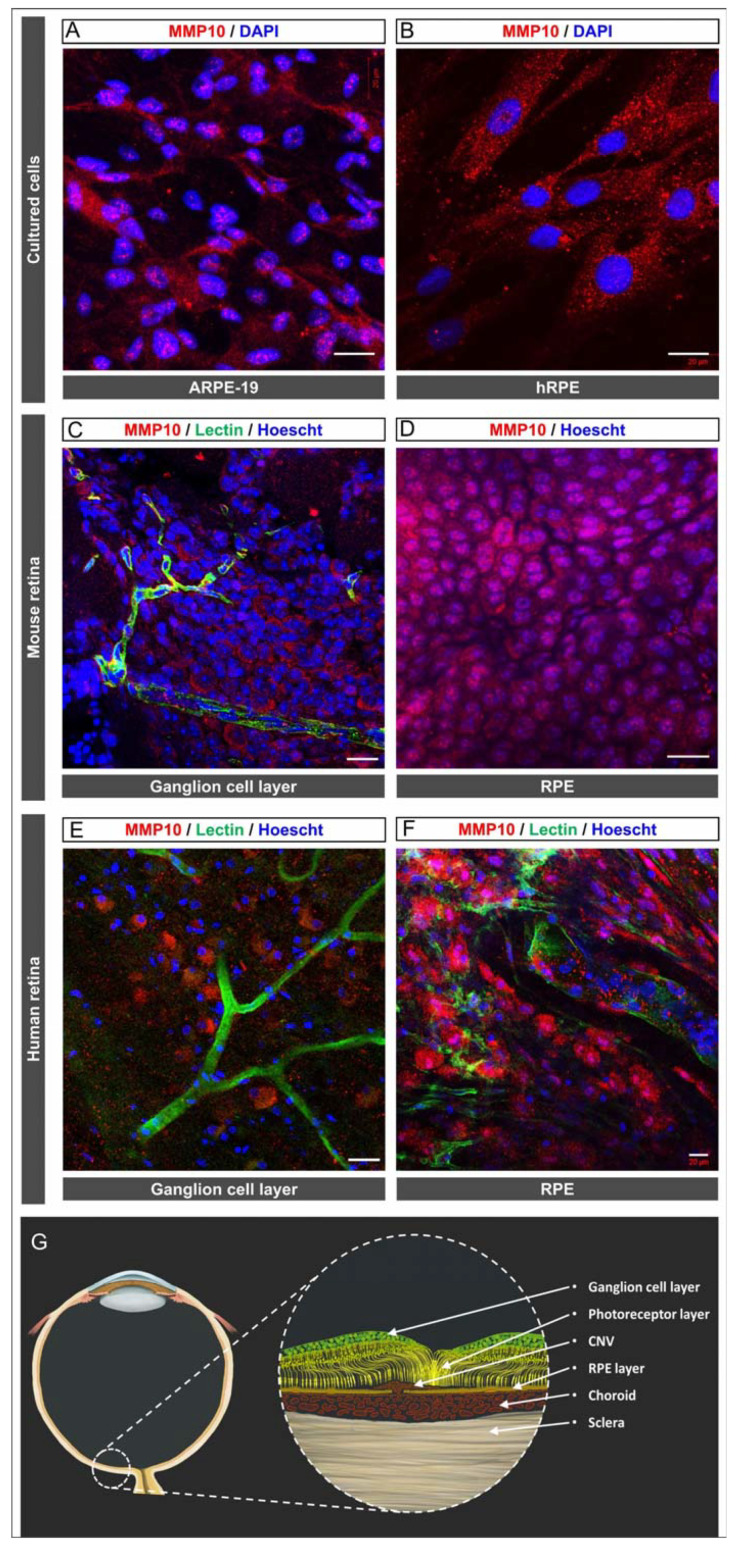
MMP10 is expressed in ARPE-19 cells, hRPE cells under basal conditions and in the control mouse and human retina. (**A**) ARPE-19 cells in transwell inserts expressing MMP10 (red). (**B**) hRPE cells expressing MMP10 (red) in coverslip. (**C**) Flat mount of WT mouse retina expressing MMP10 (red) on the RGCL. (**D**) Flat mount of WT mouse expressing MMP10 (red) on the RPE. (**E**) Flat mount of a control human expressing MMP10 (red) on the RGCL. (**F**) Flat mount of a control human retina expressing MMP10 (red) and lectin (green) on the RPE-choroid. (**G**) Human eye illustration with a magnification of the retinal in a case of CNV and the main cellular components mentioned in this manuscript, the layers mentioned in figures (**C**–**F**) are marked with an asterisk. Lectin (green) is observed in endothelial cells in vessels in mice and human retinas. DAPI and Hoescht (blue) label nuclei. CNV = Choroidal neovascularisation. MMP10 = Matrix metalloproteinase 10. RGCL = retinal ganglion cell layer. RPE = retinal pigment epithelium. Scale bar: 20 µm (**A**,**B**,**D**,**F**), 50 µm (**C**,**E**).

To determine whether ARPE-19 and hRPE cells express MMP10, immunofluorescent staining was performed on confluent ARPE-19 and hRPE cells. Under basal conditions, the cells uniformly expressed MMP10 (Figure 1A,B).

#### 3.1.2. Mice and Human RPE and Ganglion Cell Layer Expressed MMP10

Immunofluorescent staining of mice and human retinal flat mounts showed expression of MMP10 in the RPE (Figure 1D,F) and in the retinal ganglion cell layer (Figure 1C,E) in control samples.

#### 3.1.3. MMP10 Is Overexpressed in ARPE-19 Cells under Oxidative Stress Conditions

**Figure 2:**
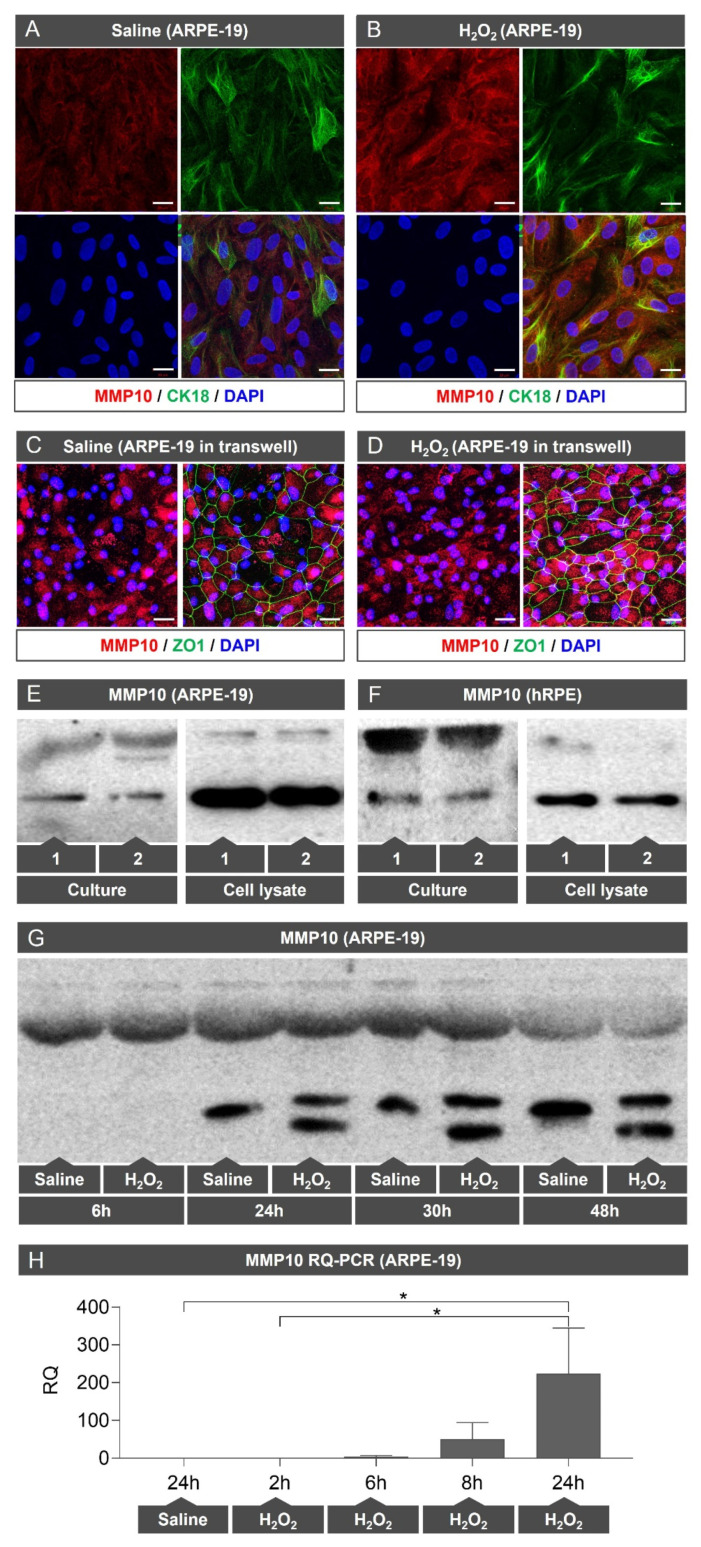
MMP10 is expressed in ARPE-19 cells and overexpressed under oxidative stress conditions. (**A**) Control ARPE-19 cells expressing MMP10 (red) and cytokeratin 18 (CK18) (green) in coverslips. (**B**) ARPE-19 cells exposed to H_2_O_2_ at 800 μM during 48 h showed a prominent increase in MMP10 expression (red) in coverslips. DAPI (blue) label nuclei. (**C**) ARPE-19 cells in transwell inserts in saline medium. (**D**) ARPE-19 cells in transwell inserts exposed to H_2_O_2_ at 800 μM during 48 h showed a prominent increase in MMP10 expression (red). (**E**,**F**) WB analysis of MMP10 under basal conditions in the supernatant and cell lysate of ARPE-19 (**E**) and hRPE (**F**) cells. As can be observed, both cell lines have a marked expression of MMP10 contained intracellularly and present a measurable concentration of extracellular MMP10. No proteolytic activation of MMP10 is observed in basal conditions, neither intra- nor extracellularly. (**G**) WB analysis of MMP10 in supernatant of ARPE-19 cells in saline medium and exposed to H_2_O_2_ at 800 μM at different times (6, 24, 30 and 48 h). MMP10 is expressed under basal conditions over time and H_2_O_2_ exposition seems to activate the protein generating two visible bands. (**H**) RT-PCR shows a significative increased in MMP10 mRNA transcription over time under oxidative stress conditions. Scale bar: 20 µm. MMP10 = Matrix metalloproteinase 10. * *p* < 0.05.

To evaluate the effect of oxidative stress, ARPE-19 cells were exposed to 800 μM H_2_O_2_ for 6, 24, 30, and 48 h. These cells showed a prominent increase in MMP10 expression, which was visible on immunofluorescence staining mainly after 48 h of oxidative stress induction (Figure 2B,D). RQ-PCR shows a significant increase in MMP10 mRNA transcription over time under oxidative stress compared to control (*p* = 0.017; Figure 2H).

#### 3.1.4. MMP10 Is Activated upon Exposure to H_2_O_2_

In addition to overexpression, protein analysis performed on ARPE-19 cells showed that proteolytic activation of MMP10 consistently generated two bands on WB (Figure 2G). This activation was not observed in cells under basal conditions (Figure 2E,F).

### 3.2. Laser-Induced Choroidal Neovascularisation in Mice

#### 3.2.1. MMP10^-/-^ Mice Develop Smaller CNV Areas after Laser Injury

**Figure 3:**
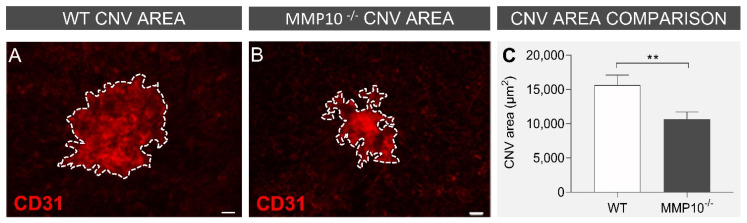
MMP10^-/-^ mice develop smaller CNV areas after laser injury. Representative laser-induced CNV area stained with CD31 by immunofluorescence (red) from WT mice (**A**) and for MMP10^-/-^ mice in RPE (**B**). The dotted lines represent the edge of the CNV area. (**C**) Comparison of mean CNV area between WT and MMP10^-/-^ mice after laser-induced CNV. AMD = age-related macular degeneration, CNV = Choroidal neovascularisation, WT = wild type, MMP10 = Matrix metalloproteinase 10. Error bars correspond to SEM. ** *p* < 0.01. Scale bar: 20 µm.

To assess the effect of MMP10 on laser-induced CNV, wild-type (WT) mouse flat mounts were compared with those of MMP10^-/-^ mice. When comparing the area of CNV delimited by CD31 staining in both groups of mice (Figure 3A,B), a statistically significant difference in lesion size was observed, with MMP10^-/-^ mice developing smaller areas of CNV than WT mice (10.642 ± 1.061 µm^2^ vs. 15.643 ± 1.460 µm^2^, *p* = 0.007; Figure 3C).

#### 3.2.2. Endothelial Cells from Laser-Induced CNV Express MMP10

**Figure 4:**
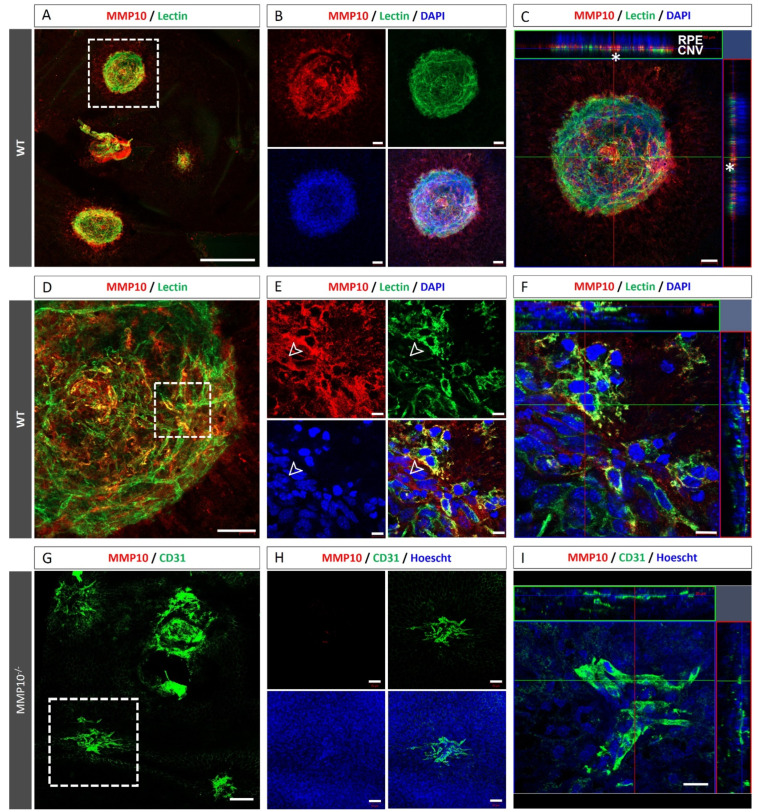
Endothelial cells (ECs) of laser-induced CNV areas are positive for MMP10 staining. Representative laser-induced CNV lesions on choroidal flat-mounts from WT mice stained with MMP10 (red), lectin or CD31 (green). (**A**) Whole flat-mount micrograph with three successfully induced CNV around the optic nerve, the outlined region represents the CNV shown in (**B**–**F**). (**B**) Magnification of dotted square in CNV area. MMP10 expression is increased in the CNV area. (**C**) Orthogonal view of the obtained z-stack showing that MMP10 expression is localized over the RPE Upper orthogonal projection showed a RPE nuclei disorganization and right orthogonal projection showed disruption in RPE nuclei (*). (**D**) Magnification of the same CNV in which the outlined region represents the magnification shown in E and F. (**E**) Magnification (63×) inside the CNV area showing that the endothelium (green) is positive for MMP10 (red). Some MMP10 positive cells are lectin negative (white arrow). (**F**) Orthogonal view of the obtained z-stack. (**G**) Whole flat-mount micrograph of a MM10^-/-^ mice with laser-induced CNV. The outlined region represents the CNV shown in H and I. (**H**) Magnification of the CNV in a MM10^-/-^ mice, as expected, no MMP10 expression is detected. (**I**) Orthogonal view of the obtained z-stack. Hoescht and 4′,6-diamidino-2-phenylindole (DAPI) (blue) label nuclei. CNV = Choroidal neovascularisation. MMP10 = Matrix metalloproteinase 10. ON = optic nerve. WT = wild type. Scale bars: 10 µm (**E**,**F**), 20 µm (I), 50 µm (**B**–**D**,**H**), 100 µm (**G**) and 500 µm (**A**).

First, we addressed whether laser-induced CNVs in WT mice expressed MMP10 by immunofluorescence staining of flat mounts and observed that MMP10 expression was mainly localised in the CNV area in the RPE (Figure 4A–F). The absence of immunofluorescence labelling for MMP10 in MMP10^-/-^ mice demonstrates the non-existence of cross-immunoreactivity with other MMPs or nonspecific labelling (Figure 4H) in these conditions. CNV endothelial cells (ECs) stained with lectin were positive for MMP10 and accounted for most of the MMP10 stained area (Figure 4D–F). However, some MMP10-negative cells were not positive for lectin, indicating that other MMP10-expressing cells may be present (Figure 4E). As expected, MMP10^-/-^ mice did not show MMP10 staining (Figure 4G–I).

### 3.3. MMP10 Plasma Levels Case-Control Study

Fifty-two patients with wet AMD were selected, including 37 females and 15 males, with a mean age of 72.70 ± 0.99 years. Fifty-two age- and sex-matched controls with a mean age of 72.61 ± 0.98 years were selected in a 1:1 ratio. As expected, no statistically significant differences in age were found between the groups (*p* = 0.951), with a mean age difference between matches of 0.9 ± 0.15 years. The other demographic characteristics were also not significantly different between the groups (Table 1).

**Figure 5:**
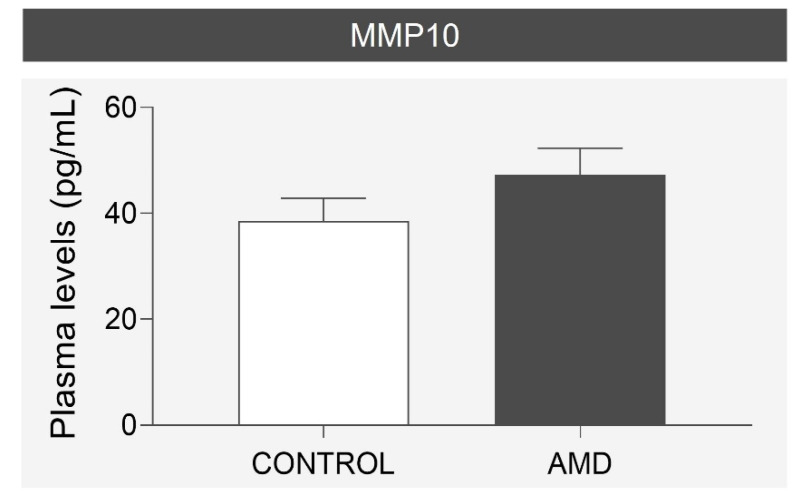
Comparison of mean MMP10 plasma levels in the age- and sex-matched case-control cohort. No statistically significant differences were found. AMD = age-related macular degeneration, MMP10 = Matrix metalloproteinase 10.

Although patients with wet AMD showed a slight increase in plasma MMP10 levels, no statistically significant difference was observed between the groups (38.52 ± 4.34 vs. 47.26 ± 5.03 pg/mL, *p* = 0.283) (Figure 5).

**Figure 6:**
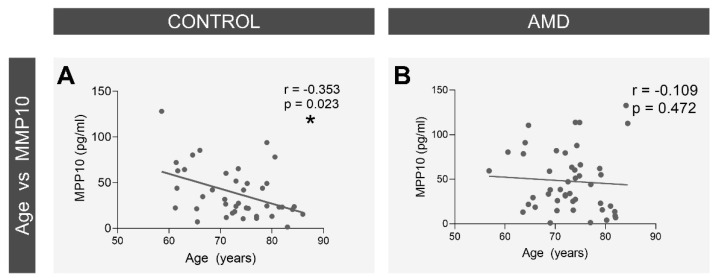
Correlation of age with the mean plasma levels of MMP10. A statistically significant negative correlation was observed in the control group (**A**) while no apparent correlation was found in the AMD group (**B**). AMD = Age-related macular degeneration, MMP10 = Matrix metalloproteinase 10. The Pearson’s r values are displayed on each graph along with the *p* value of statistical significance. * *p* < 0.05.

Analysis of the correlation between variables and age (Figure 6) revealed that the plasma levels of MMP10 significantly decreased with age in the control group (r = −0.353, *p* = 0.023); however, no statistically significant correlation was found in the AMD group (r = −0.109, *p* = 0.472).

## 4. Discussion

In this study, we found an interesting and novel link between MMP10 and CNV. ARPE-19 and human RPE cells release MMP10, which is activated under oxidative stress conditions. Moreover, we demonstrated the involvement of MMP10 in the development of CNV in mice for the first time. MMP10-knockout mice developed smaller areas of neovascularisation than WT mice, revealing a clear association of MMP10 with this process. In addition, it was shown that the increase in MMP10 is a local phenomenon limited to the neovascular membrane that does not modify the plasma concentration of this protein.

In the early stages of AMD, choriocapillaris dropout [20], oxidative stress [6], and a pro-inflammatory state [21] promote an environment in which the combination of stimulatory signals released by the retina, RPE, and choroid prompts remodelling of the vasculature. In ARPE-19 cells, oxidative stress conditions (as observed in AMD) could activate MMP10. Released soluble growth factors, chemokines, and cytokines activate some quiescent ECs, transforming them into tip cells, the leading cells at the tips of vascular sprouts [22]. The capillary basement membrane, mainly composed of type IV collagen, heparin-sulfated proteoglycans, entactin, and laminin [23], is the first barrier that these cells encounter. MMP10 can degrade all of these components [24]. We found that CNV ECs expressed MMP10 in mice. Moreover, vascular endothelial growth factor (VEGF), the main regulatory factor of angiogenesis [25], has been shown to trigger the expression and synthesis of MMP10 by ECs, enhancing their proteolytic capacity [26] and in histone deacetylase 7 (HDAC7)-knockdown HUVECs [27]. VEGF itself is influenced by the activity of MMPs as some of the different isoforms are sequestered in the ECM and are unable to diffuse freely, but they can be cleaved by MMPs secreted by the sprout tips [28]. The release of this sequestered VEGF regulates vascular patterning and can transform normal angiogenesis into a pathological process [29]. In addition to merely degrading the basement membrane, proteolytic cleavage of collagen type IV results in exposure of cryptic binding sites hidden in the triple helical structure that facilitates EC adhesion and migration and is required for angiogenesis in vivo [30]. Blocking these cryptic sites inhibits retinal angiogenesis in vivo [28].

After passing through the basement membrane, the sprouting vessels must cross the BM. Metalloproteinases can degrade all components of the BM; therefore, they have a dual role. On the one hand, they can facilitate the renewal of BM in a process of continuous cycles of synthesis and degradation, thus preventing the accumulation of abnormal ECM material and allowing a proper exchange of nutrients and waste products. On the other hand, excessive degradative activity may affect the integrity of the BM and allow the progression of neovessels [31]. The expression of proteases in the RPE leads to degradation of elastin in the choroid mimicking features of polypoidal choroidal vasculopathy, a common subtype of neovascular AMD [32]. We have previously reported that the RPE, on which the BM underlies, expresses MMP10. Because MMPs require a proteolytic activation process, their activation can be studied by the differentiation of the proenzyme from the active form with lower molecular weight on WB. Good correlation has been reported between the proteolytic activity of MMP10 and presence of a second band of lower molecular weight on WB [33]. In our study, we found that MMP10 was activated under conditions of oxidative stress in the RPE. In this regard, it has been shown that reducing intracellular oxidative stress, both by transfecting cells with a physiological redox regulator [34] and by adding an antioxidant [35], β-Carotene, reduces MMP10 expression in cells. Based on these findings, it can be speculated that MMP10 may have either a beneficial role in facilitating the recycling of the BM in situations where its deterioration increases cellular stress or a deleterious role in facilitating the sprouting vessels to cross the membrane.

The animal model of laser-induced CNV that we have used in this study triggers neovascularisation from a rupture in the BM and is therefore useful to assess whether MMP10 plays a role in the development of CNV once ECs overcome the basement membrane and BM. MMP10-deficient mice develop smaller neovascular membranes; therefore, MMP10 favours the development of CNV beyond the rupture of basement membranes or the BM.

Notably, MMP10 has also been implicated in the pathophysiology of atherosclerosis and diabetic retinopathy [16]. MMP10 overexpression has been described as compromising vascular integrity and to be associated with aortic aneurysms and atherosclerotic lesions [17,36]. In fact, increased plasma levels of MMP10 are associated with more severe atherosclerosis [37].

Given the various functions exerted by MMPs, their possible role as biomarkers has been studied in several pathologies. Most of these studies have been conducted in the field of oncology, with a special focus on MMP1, MMP2, and MMP9, and have shown potential benefits in early detection and evaluation of disease progression, prognosis, and metastasis [38]. Nevertheless, MMPs have also been widely explored in inflammatory disorders [39] and cardiovascular pathology [40]. However, the use of MMPs as potential biomarkers in AMD has not been extensively studied. Chau et al. found significantly higher plasma levels of MMP9 in the CNV group than in the control group [41]. Renpan Zeng et al. obtained similar results regarding the serum levels of MMP9 and found that MMP2 levels were significantly higher in AMD patients [42]. Krogh-Nielsen et al. reported elevated plasma concentrations of MMP9 in patients with AMD and geographic atrophy [11]. However, there may be differences between systemic expression levels and local expression of these proteins and plasma measurements may reflect a systemic imbalance in cells involved in pathogenesis, such as ECs or circulating immune cells. Circulating and systemic immune cells have been shown to be involved in the late stages of AMD in observational studies [43,44], and MMP10 is expressed by inflammatory cells such as macrophages [45]. In this study, we explored the plasma concentration of MMP10 in neovascular AMD in an age- and sex-matched case-control cohort and found no significant differences between the groups demonstrating that the elevation of MMP10 in the retina does not correlate with an elevation in plasma levels, suggesting that the overexpression occurs locally and is a consequence of the neovascularisation process rather than a systemic imbalance. Previous studies reaffirm this fact in which the local concentration of MMP10 in the vitreous of patients with ischemic central retinal vein occlusion is increased while at the serum level a reduction in its concentration is observed [18]. The same has been described in the lung, where serum levels of MMP10 in patients with idiopathic pulmonary fibrosis are low in contrast to the abundant expression of MMP10 in lung tissue [46].

It is commonly accepted that the aetiology of AMD is multifactorial, with age being the most important risk factor [47]. Thus, it would be interesting to study whether there is a correlation between MMP expression and age. In our control group, we observed a decrease in MMP10 levels with age, this observation argues against an increase in MMP10 activity with age as a trigger for the disease. However, in the AMD group, MMP10 was not correlated with age despite the same sample size. These results suggest that in patients with AMD, MMP10 levels do not decrease as much with age or that the cellular environment in these patients favours an increase in its expression (as observed in CNV in mice) that counteracts the age-related decrease.

The strength of our research is that we have studied the role of MMP10 in AMD from multiple approaches: from a cellular point of view, in an animal neovascular AMD model, in human donor eyes, and in human plasma samples. This is the first time that MMP10 has been implicated in the pathophysiology of AMD.

However, our study has some limitations. A larger sample size would help gain statistical power and provide greater reliability to the results in the future. Further, plasma levels of MMP10 may differ from tissue level concentrations (in the retina, RPE, and choroid); therefore, relationships with the pathophysiology of AMD should be cautiously established. Nevertheless, obtaining intraocular tissue is a complex invasive process, in contrast with the ease and safety of obtaining plasma samples.

In future studies we purposed some objectives. Firstly, it would be interesting to assess the effect of antioxidants on MMP10 expression and activation in RPE cells and mouse retina. It would also be interesting to evaluate the effect of intravitreal MMP10 inhibitors as antiangiogenic therapy.

Secondly, a growing body of literature reflects that MMP10 has a role in angiogenesis. It has been shown in vitro in HUVEc cells that siRNA inhibition of MMP10 inhibited migration and tube formation [26], equivalent results were observed in these same cells when adding as culture medium the supernatant of Hela cells in which MMP10 was overexpressed or silenced [48]. Increasing MMP10 expression stimulates the expression of HIF-1α and MMP2 (pro-angiogenic factors) and PAI-1 and CXCR2 (pro-metastatic factors), and accordingly, targeting MMP10 with siRNA in vivo resulted in diminution of xenograft tumor growth with a concomitant reduction of angiogenesis and a stimulation of apoptosis [48]. Moreover, in animal models of induced hepatocarcinoma [49] MMP10 KO mice developed reduced tumor vascular density, as measured by CD31 immunostainings, compared to control mice. Subsequently, it would be very interesting to further examine the in vitro relationship between MMP10 and choroidal neovascularization adding the supernatant of oxidative-stressed ARPE-19 cells in an in vitro angiogenic assay model with choroidal endothelial cells in order determine the effect of MMP10 in the migratory response of endothelial cells.

## 5. Conclusions

Our study shows cellular, animal, and human evidence regarding the relationship between MMP10 and the development of neovascular AMD and specifically the development of CNV. Our experiments demonstrate that MMP10 is expressed by the RPE, overexpressed, and activated under oxidative stress conditions. MMP10^-/-^ mice develop smaller neovascular membranes, reflecting its involvement in the CNV process. Furthermore, plasma MMP10 concentrations do not differ between patients with neovascular AMD and control patients, demonstrating that MMP10 induction is a local phenomenon. These preliminary data demonstrate that MMP10 plays a role in choroidal neovascularisation and its usefulness as a potential new therapeutic target for neovascular AMD.

## Figures and Tables

**Table 1:** Demographic data and clinical characteristics of participants.

	Control (*n* = 52)	AMD (*n* = 52)	*p*-Value
**Age (years)**	72.70 ± 0.99	72.61 ± 0.98	0.951
**Female (%)**	71%	71%	1.0
**HBP (%)**	33%	44%	0.314
**Smokers (%)**	17%	25%	0.326
**Dyslipidemia (%)**	27%	34%	0.524
**Cardiovascular diseases (%)**	19%	19%	1.0

AMD = Age-related macular degeneration. HBP = High blood pressure.

## Data Availability

All data are contained within the article.
